# Direct Binding of the pH-Regulated Protein 1 (Pra1) from *Candida albicans* Inhibits Cytokine Secretion by Mouse CD4^+^ T Cells

**DOI:** 10.3389/fmicb.2017.00844

**Published:** 2017-05-11

**Authors:** Arne Bergfeld, Prasad Dasari, Sandra Werner, Timothy R. Hughes, Wen-Chao Song, Peter Hortschansky, Axel A. Brakhage, Thomas Hünig, Peter F. Zipfel, Niklas Beyersdorf

**Affiliations:** ^1^Institute for Virology and Immunobiology, University of WürzburgWürzburg, Germany; ^2^Department of Infection Biology, Leibniz-Institute for Natural Product Research and Infection Biology, Hans-Knöll-InstituteJena, Germany; ^3^Friedrich Schiller UniversityJena, Germany; ^4^Complement Biology Group, Division of Infection and Immunity, Cardiff School of Medicine, and the School of Biosciences, Cardiff UniversityCardiff, UK; ^5^Centre for Experimental Therapeutics and Department of Pharmacology, University of Pennsylvania School of Medicine, PhiladelphiaPA, USA; ^6^Department of Molecular and Applied Microbiology, Leibniz Institute for Natural Product Research and Infection Biology, Hans Knoell InstituteJena, Germany

**Keywords:** *Candida albicans*, ph-regulated antigen 1 (Pra1), CD4^+^ T cells, immune evasion, cytokine secretion

## Abstract

Opportunistic infections with the saprophytic yeast *Candida albicans* are a major cause of morbidity in immunocompromised patients. While the interaction of cells and molecules of innate immunity with *C. albicans* has been studied to great depth, comparatively little is known about the modulation of adaptive immunity by *C. albicans*. In particular, direct interaction of proteins secreted by *C. albicans* with CD4^+^ T cells has not been studied in detail. In a first screening approach, we identified the pH-regulated antigen 1 (Pra1) as a molecule capable of directly binding to mouse CD4^+^ T cells *in vitro*. Binding of Pra1 to the T cell surface was enhanced by extracellular Zn^2+^ ions which Pra1 is known to scavenge from the host in order to supply the fungus with Zn^2+^. *In vitro* stimulation assays using highly purified mouse CD4^+^ T cells showed that Pra1 increased proliferation of CD4^+^ T cells in the presence of plate-bound anti-CD3 monoclonal antibody. In contrast, secretion of effector cytokines such as IFNγ and TNF by CD4^+^ T cells upon anti-CD3/ anti-CD28 mAb as well as cognate antigen stimulation was reduced in the presence of Pra1. By secreting Pra1 *C. albicans*, thus, directly modulates and partially controls CD4^+^ T cell responses as shown in our *in vitro* assays.

## Introduction

*Candida albicans* is a commensal on human skin and mucosal surfaces. In situations of immunosuppression, *C. albicans* may, however, become pathogenic. Prominent examples of *C. albicans*-induced pathologies are mucosal or skin candidiasis as well as *C. albicans* septicemia in ICU and/ or HIV/Aids patients (Klein et al., 1984; Sangeorzan et al., 1994; Leroy et al., 2009). In the latter cohorts, loss of CD4^+^ T cells is the hallmark of immunodeficiency. This highlights the importance of CD4^+^ T cells for controlling *C. albicans* infections in humans.

To allow commensalism, *C. albicans* has evolved a number of evasion strategies to protect itself from attack by the host’s immune system (Zipfel et al., 2011). Immune evasion might be beneficial during commensal growth as it avoids potentially harmful inflammation and adaptive immune responses. The very same mechanisms might, however, contribute to *C. albicans* pathogenicity once epithelial barriers are disturbed. Research into the factors driving *C. albicans* pathogenicity led to the discovery of the pH-regulated antigen 1 (Pra1) as a multifaceted immune evasion protein (Zipfel et al., 2011). Pra1 interferes with innate immunity including the complement cascade on different levels thereby efficiently protecting the fungus from complement attack. Moreover, Pra1 scavenges zinc from the host, thus, ensuring sufficient supply of the fungus with this bivalent cation (Citiulo et al., 2012). For both functions, complement inhibition and zinc scavenging, Pra1 is first secreted, interacts with complement proteins or zinc in solution and then the complex of Pra1 and its binding partner are recruited back to the *C. albicans* surface (Zipfel et al., 2011; Citiulo et al., 2012).

As Pra1 is secreted by *C. albicans* we hypothesized that this fungal protein might also be capable of bypassing fungal sensing by DCs (Romani, 2011) and of directly interacting with CD4^+^ T cells, thus, modulating T cell function in its favor. Having established that recombinantly expressed Pra1 binds to mouse CD4^+^ T cells, we, thus, analyzed the impact of Pra1 on T cell activation, expansion and effector cytokine secretion. Our data suggest that *C. albicans* directly modulates anti-fungal immunity through secreting T cell-binding proteins like Pra1.

## Materials and Methods

### Mice

Wild-type C57BL/6J mice and OT-II C57BL/6J mice (Barnden et al., 1998) were bred in the animal facility of the Institute for Virology and Immunobiology, University of Würzburg. CD55^-/-^ C57BL/6 mice (Sun et al., 1999) were obtained from the University of Cardiff and also bred in our animal facility. Crry^-/-^ C57BL/6 (Ruseva et al., 2009) and CD59a^-/-^ C57BL/6 mice (Holt et al., 2001) were bred at Cardiff University. All mice were kept in a specified pathogen free conventionally housed environment and used for experiments between six and 21 weeks of age.

### Antibodies and Flow Cytometry

The following antibodies and reagents were used to stain mouse cells: anti-CD4 Alexa Fluor 647 (clone RM4-5), anti-IFNγ Alexa Fluor 488 (clone XMG1.2), Streptavidin-PerCP (all BioLegend, San Diego, CA, USA), anti-CD25 biotin (clone 7D4, BD Pharmingen, Franklin Lakes, NJ, USA) anti-CD55 unconjugated (RIKO-3, Biolegend), anti-CD11b FITC (clone M1/70), anti-B220 Alexa Fluor 647 (clone RA3-6B2) (all BD Pharmingen), anti-CD3 PerCp (clone 145-2C11, BioLegend).

For staining of Pra1 a polyclonal antibody was raised in rabbits by immunization with purified recombinant Pra1. Aspf2-antiserum was generated by immunization of mice with purified recombinant Aspf2. Secondary polyclonal antibodies for staining of primary antibodies were goat anti-mouse-Ig FITC and donkey anti-rabbit-Ig PE (Jackson Immunoresearch, West Grove, PA, USA). Flow cytometry was performed on a FACSCalibur or LSR II flow cytometer using either CellQuest or DIVA software (BD Bioscience, Franklin Lakes, NJ, USA). We used FlowJo (TreeStar) to further analyze FACS data.

### Protein Expression and Purification

Recombinant Pra1wt and Aspf2 were expressed in *Pichia pastoris* and isolated *via* the His-tag (Luo et al., 2009; Bacher et al., 2014). For protein overexpression and purification, the *pra1* gene encoding a protein lacking the C-terminal 61-amino acid was amplified from the pPICZαB-Pra1wt clone using the sequence specific forward primers ACTGAATTCTGTGGAGCCATCCGCAGTTTGAAAAAAGCGCGGCACCAGTTACGGTTACC and reverse primer ACT*GGTACC*GCGCACCCTTCGCCGGGAATTG, containing the restriction sites *Eco*RI and *Kpn*I. The PCR product and plasmid pPICZαB were enzymatically digested, ligated, and sub-cloned into pPICZαB (Invitrogen, Karlsruhe, Germany). The resulting plasmid pPICZαB-Pra1ΔC61 was transfected and overexpressed in *Pichia pastoris* X33 (EasySelect^TM^Pichia Expression Kit, Invitrogen, Karlsruhe, Germany). The Pra1ΔC61 was purified as described (Luo et al., 2009).

### Organ Processing and FACS Stainings

Single cell suspensions were generated by mashing cervical, axillary, inguinal and mesenteric lymph nodes or spleens through a cell strainer (Falcon, Pittsburg, PA, USA). Single cell suspensions of splenocytes were then subjected to red cell lysis by hypoosmotic shock. Lymph node and red cell-lysed spleen cells were then resuspended in buffered salt solution (BSS) containing 0.1% (w/v) bovine serum albumin (BSA). Total lymph node or spleen cells were incubated with Pra1 (10 μg/ ml) or Aspf2 (10 μg/ ml) in PBS at 37°C for 30 or 45 min. For investigation of the influence of zinc on Pra1 binding, ZnCl_2_ (1, 10, or 100 μM) was added while incubating cells together with Pra1. After washing bound Pra1 or Aspf2 were detected with a polyclonal anti-Pra1- (rabbit) or anti-Aspf2- (mouse) antiserum followed by PE anti-rabbit Ig polyclonal antibody (donkey; Dianova) or FITC anti-rabbit Ig polyclonal antibody (donkey; Dianova). For further stainings the samples were blocked with normal rabbit serum (1:500) or normal mouse Ig (20 μg/ ml, Sigma) followed by incubation with anti-CD4 mAb (Alexa Fluor 647) alone or together with anti-CD3 mAb (PerCp). For Kv1.3 detection, ShK-F6CA (0.3 μg/ml; Bachem AG, Bubendorf, Switzerland) was incubated together with mAb against cell surface proteins for 30 min at room temperature (Beeton et al., 2003).

### Polyclonal Stimulations *In Vitro*

To test for co-stimulation lymph node cells from WT mice were first enriched for CD4^+^ T cells (MagniSort Mouse CD4 T cell Enrichment Kit, eBioscience, Santa Clara, CA, USA or CD4^+^ T cell isolation kit, Miltenyi) resulting in at least 93% pure CD4^+^ T cells. Afterwards the cells were stained with anti-CD4-Alexa Fluor 647 and CD4^+^ T cells sorted using the FACS Aria III (BD) cell sorter (100% purity). For analysis of cell proliferation cells were incubated for 5 min at RT with 5 μM Vybrant CFDA SE Cell Tracer Kit (CFSE, Life Technologies, Carlsbad, CA, USA). Anti-CD3-mAb (2.5 μg/ ml, clone 145-2C11, BioLegend) was bound to 96-flat bottom-plates (Greiner, Kremsmuenster, Austria) after incubation on the plate o/n at 4°C dissolved in 0.1 M NaHCO_3_-buffer (pH 9). After coating of the plate, non-specific binding was blocked by incubation with normal mouse immunoglobulin (20 μg/ml in BSS/0.1% BSA (w/v), Sigma Aldrich, St. Louis, MO, USA) at 37°C for 30 min. 1 × 10^5^ CFSE-labeled CD4^+^ T cells were added per well and anit-CD28 mAb E18 (Exbio) (Dennehy et al., 2006) (1 and 10 μg/ ml) or Pra1 (0.1 pg/ ml – 100 ng/ ml) were added in solution. For the cultures, we used complete RPMI 1640 medium supplemented with 1 mM sodium pyruvate, non-essential amino acids MEM (0.05–2 mM), 100 U/ ml penicillin and 100 μg/ml streptomycin, 30 μM mercaptoethanol, 2 mM L-glutamine (all Gibco) and 10% (v/v) heat-inactivated fetal calf serum. After 3 days, CD4 and CD25 were stained and expression of both markers, together with CFSE dilution, was analyzed by flow cytometry. To determine cytokine secretion, magnetically purified CD4^+^ T cells were cultured with plate-bound anti-CD3 mAb in the presence of soluble Pra1 (1–100 ng/ml) or anti-CD28 mAb (10 μg/ml) as already described. Alternatively, we coated Dynabeads^®^ Pan Mouse IgG (Invitrogen) with 10 μg/ml anti-CD3 mAb (clone 145-2C11, BioLegend) and 1 μg/ml anti-CD28 mAb (clone E18) according to the manufacturer’s instructions and added the beads at a bead to cell ratio of 5:1 to the cultures. After three days, culture supernatants were harvested and frozen at –70° C for subsequent cytokine analysis.

### Stimulation of OT-II CD4^+^ T Cells *In Vitro*

Lymph node cells and red blood cell-lysed splenocytes from OT-II mice were pooled and 2 × 10^5^ cells seeded per well of a 96-well round bottom plate (Greiner). OVA 323-339 peptide (OVAp, Charité Berlin) was added at 1 or 0.1 μM. For each condition six technical repeats were set up. On day three, culture supernatant was harvested and frozen at –70°C. Cells were stained for CD4 and CD25 expression and absolute cell numbers were determined by FACS using counting beads. To generate Th1 cells pooled spleen/lymph node cells from an OT-II mouse were depleted of CD4^+^ CD25^+^ regulatory T cells using anti-CD25 biotin (5 μg/ml, BD) and Streptavidin-beads and passage over an LD column (both Miltenyi). CD25-depleted spleen/lymph node cells were then seeded at 1 × 10^6^ cells/well (48-well plate, Greiner, final volume: 1 ml, 3 × 10^6^ cells in total), OVAp was added at 1 μM, recombinant mouse IL-12 (R&D Systems, Minneapolis, MN, USA) at 10 ng/ml and goat-anti-mouse IL-4 (R&D Systems) at 10 μg/ml. To obtain ‘Th0’ cells the CD25-depleted spleen/lymph node cells were stimulated with OVAp only. After five days of culture, CD4^+^ T cells were magnetically purified (CD4^+^ Isolation kit, Affymetrix, according to manufacturer’s instructions) and cultured for another two days in the presence 0.1 μM Proleukin^®^ (Novartis) (48-well plate, 5 × 10^5^ cells/well). Afterward, the cells were harvested and co-cultured with T cell-depleted splenocytes (anti-CD90.2 beads, LD column, Miltenyi) isolated from a WT C57BL/6 mouse (96-well round-bottom plate; 1 x 10^5^ T cell-depleted splenocytes per well, 1 × 10^4^ Th1 cells per well, triplicates). OVAp and Pra1 were added to these cultures in different concentrations. Every day 15 μl of culture supernatant were harvested per well to determine cytokine concentrations. On day three the Th1 cells were restimulated with phorbol myristate acetate (5 ng/ml) and ionomycin (500 ng/ml) for two hours at 37°C/5% CO_2_ (v/v) before addition of Brefeldin A (10 μg/ml; all Sigma) and incubation for another two hours followed by cell surface staining for CD4, fixation, and permeabiliziation of the cells (Fix/Perm and Perm buffers, ebioscience) and intracellular staining for IFNγ expression (anti-IFNγ Alexa Fluor 488, clone XMG1.2, Biolegend).

### Cytokine Detection in Culture Supernatants

Concentrations of the indicated cytokines were determined in culture supernatants using LEGENGplex^TM^ (Biolegend) according to the manufacturer’s instructions.

### Statistics

Summary graphs were generated and statistical testing was done using Excel © 14.4.1 (Microsoft) and Prism 4.0c © (GraphPad). *P* < 0.05 was considered statistically significant.

### Ethics Statement

Stadt Würzburg (City of Würzburg) and UK Home Office (PPL 30/3038) approved breeding of the mice used in this study and the animals were culled by Annex IV approved techniques in accordance with Directive 2010/63/EU.

## Results

### Pra1 Directly Binds to Mouse CD4^+^ T Cells in a Zinc-Dependent Manner

**FIGURE 1:**
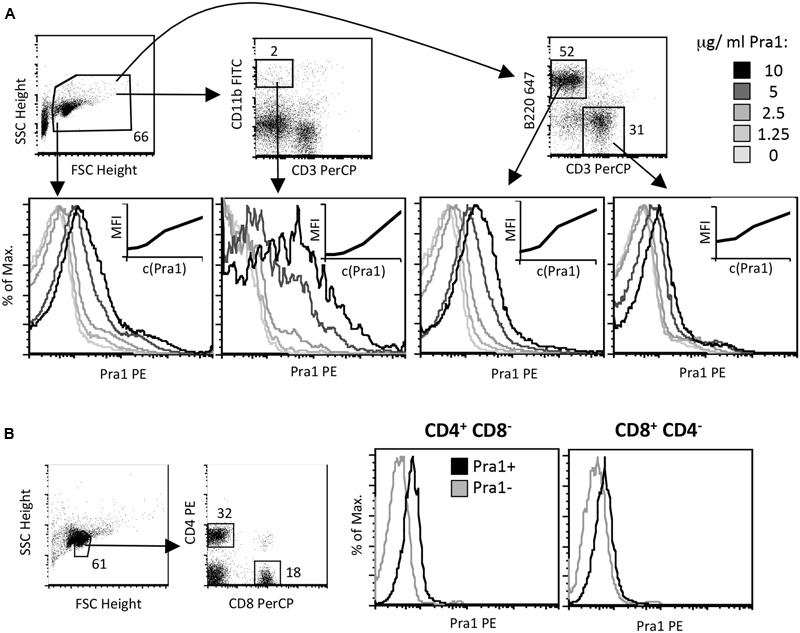
***Candida albicans* Pra1 binds to monocytes, B and T cells. (A)** Total mouse splenocytes were incubated with Pra1 in the absence of exogenously added zinc. Pra1 binding was detected indirectly as described in Section “Materials and Methods”. Pra1 bound to all splenocytes (left histogram), CD11b^+^ CD3^-^ monocytes (middle left histogram), B220^high^ CD3^-^ B cells (middle right histogram) and T cells (right histogram) dose-dependently. **(B)** Total mouse lymph node cells were gated for CD4^+^ and CD8^+^ T cells as depicted and Pra1 binding to these subpopulations determined (in the absence of exogenously added zinc). **(A,B)** One of at least two experiments with similar result is shown.

As Pra1 expression of *C. albicans* is induced upon contact with human cells and as it has already been shown to strongly modulate innate immunity (Zipfel et al., 2011), we studied direct binding of Pra1 to mouse CD4^+^ T cells *in vitro.* We used recombinantly expressed Pra1 purified from *Pichia pastoris* for staining and found that Pra1 bound to all splenocytes in a dose-dependent manner (**Figure 1A**, left histogram). Among total splenocytes CD11b^+^ CD3^-^ monocytic cells bound Pra1 particularly well (**Figure 1A**, middle left histogram), which was expected as complement receptor 3 (CR3, Mac1, CD11b/CD18) had been identified as a cellular receptor for Pra1 on mouse leukocytes (Soloviev et al., 2007, 2011). Splenic B (**Figure 1A**, middle right histogram) and T cells (**Figure 1A**, right), i.e., CD4^+^ and CD8^+^ T cells (**Figure 1B**), also clearly bound Pra1, albeit to a lesser extent than the monocytic cells.

**FIGURE 2:**
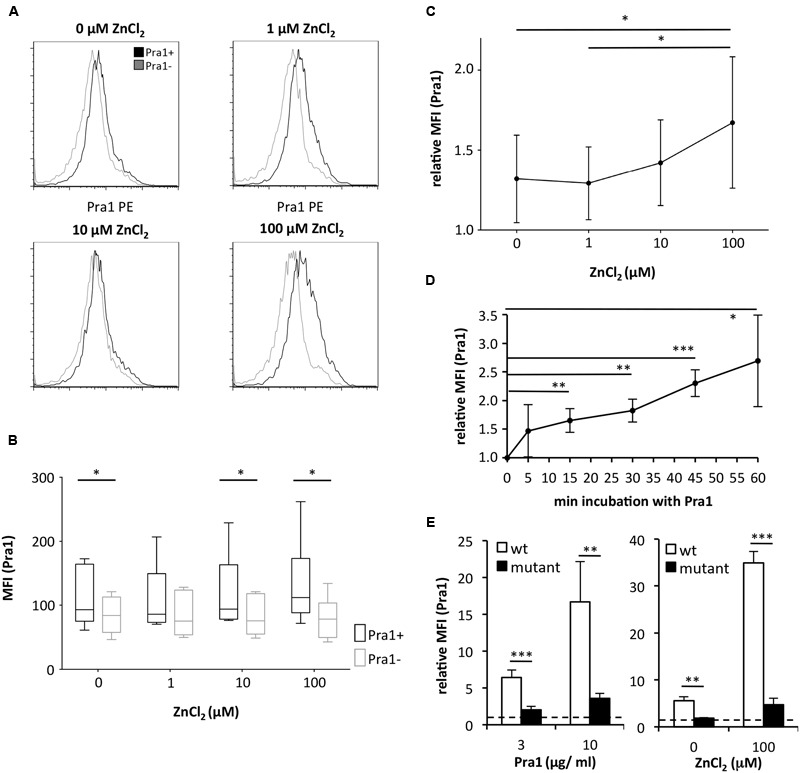
**Binding of *C. albicans* Pra1 to mouse CD4^+^ T cells is enhanced by extracellular zinc. (A)** Representative histograms depicting Pra1 binding to gated mouse CD4^+^ T cells (total lymph node cells) in the presence of different concentrations of ZnCl_2_ (black: Pra1; gray: background staining control). **(B)** Summary graph depicting Pra1 (black) versus background staining (gray) of CD4^+^ T cells in the presence of different concentrations of ZnCl_2_ (*n* = 6, two-sided *t*-test, ^∗^*p* < 0.05). **(C)** Summary graph showing enhanced binding of Pra1 to mouse CD4^+^ T cells in the presence of ZnCl_2_ (relative MFI(Pra1) = MFI(Pra1)/MFI(background); means ± SD; *n* = 6; two-sided *t*-test, ^∗^*p* < 0.05). **(D)** Pra1 on-kinetics at 37°C. Lymph node cells were incubated with Pra1 for the indicated periods of time before we detected binding of Pra1 to CD4^+^ T cells (means ± SD of *n* = 3 separate experiments; two-sided *t*-test). **(E)** Comparison of Pra1wt (white columns) and a mutated Pra1 lacking the zinc-binding domain (aa 238–299, black columns) binding to gated CD4^+^ T cells in the presence of 10 μM ZnCl_2_ (left) or 0 and 100 μM ZnCl_2_ (right). Means + SD (*n* = 3–6 individual measurements). Two-sided *t*-test. ^∗^*p* < 0.05, ^∗∗^*p* < 0.01, ^∗∗∗^*p* < 0.001.

As Pra1 binds zinc (Citiulo et al., 2012) we tested whether zinc influences Pra1 binding to mouse CD4^+^ T cells. Zn^2+^ which is found in serum at a concentration of 10 μM (Feske et al., 2012) and beyond increased Pra1 binding to mouse CD4^+^ T cells (**Figures 2A–C**) with plateau levels of binding reached after 30 min of incubation (**Figure 2D**). Moreover, a *pra1* deletion mutant encoding a Pra1 protein lacking the putative zinc-binding domain (Pra1 Δ238–299) (Citiulo et al., 2012) showed almost no binding to mouse CD4^+^ T cells (**Figure 2E**).

**FIGURE 3:**
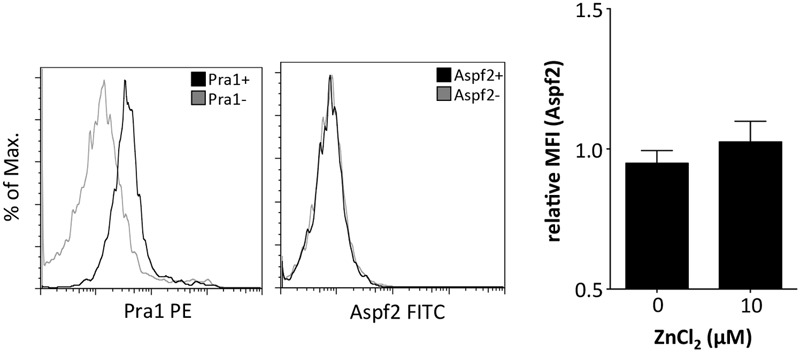
**The Pra1 homolog of *A. fumigatus*, Aspf2, does not bind to mouse CD4^+^ T cells.** Representative staining of Aspf2 on gated CD4^+^ T cells derived from the spleen as performed before with Pra1. Aspf2- and Pra1-staining on cells from the same mouse with Pra1- and Aspf2- (black) and background staining (gray). The bar diagram shows relative MFI of the Aspf2-signal with and without ZnCl_2_ (relative MFI(Aspf2) = MFI(Aspf2)/MFI(background); means ± SD; *n* = 3).

The zinc binding capacity of Pra1 is shared by its homolog in *A. fumigatus*, i.e., the Aspf2 protein (Citiulo et al., 2012). We, therefore, used recombinantly expressed (*P. pastoris*) and purified Aspf2 and tested whether Aspf2 also directly binds to mouse CD4^+^ T cells. Aspf2, in contrast to Pra1, however, did not bind to the mouse T cells even when ZnCl_2_ was added to the buffer (**Figure 3**). Thus, Pra1, but not Aspf2, directly binds to mouse CD4^+^ T cells and Pra1 binding is enhanced in the presence of extracellular zinc.

### Complement Regulatory Proteins Expressed by Mouse CD4^+^ T Cells Do Not Interact with Pra1

**FIGURE 4:**
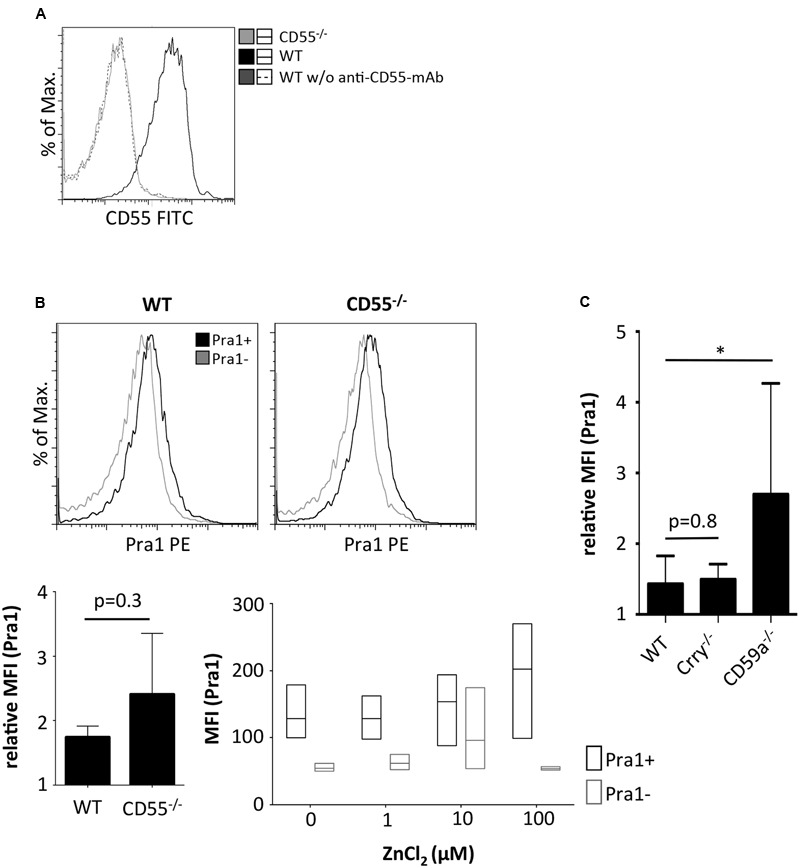
**Comparison of Pra1 binding to CD4^+^ T cells of wild-type and CD55^-/-^ mice. (A)** CD55 staining of lymphocytes from WT (black) and CD55^-/-^ mice (gray). As a staining control, WT lymphocytes were incubated without the anti-CD55 primary mAb (dashed dark gray). **(B)** Binding of Pra1 to lymph node CD4^+^ T cells from WT and CD55^-/-^ mice. Gray: Staining control without primary mAb. Left bar diagram show relative MFI of Pra1 signal in WT and CD55^-/-^ cells (relative MFI(Pra1) = MFI(Pra1)/MFI(background); means ± SD; *n* = 3; two-sided *t*-test). Right diagram depicts MFI of Pra1 staining of CD55^-/-^ CD4^+^ T cells in the presence of different concentrations of ZnCl_2_ (mean, upper and lower quartile, field of values, *n* = 3). **(C)** Comparison of Pra1 binding to splenic CD4^+^ T cells of WT, Crry^-/-^ and CD59a^-/-^ mice (*n* = 3-8 mice/group; means ± SD; two-sided *t*-test; ^∗^*p* < 0.05).

So far, only complement receptor 3 (CR3, Mac1, CD11b/CD18) has been identified as a cellular receptor for Pra1 on mouse leukocytes (Soloviev et al., 2007, 2011). As the staining pattern of Pra1 showed that Pra1 binds similarly well to all mouse CD4^+^ T cells (**Figure 1B**) we hypothesized that a complement regulatory protein expressed by all mouse T cells might be the receptor for Pra1. Therefore, we analyzed Pra1 binding to CD4^+^ T cells of CD55^-/-^ mice in more detail as CD55, Crry, and CD59a are the three complement-regulatory proteins expressed by mouse T cells (Miwa and Song, 2001). While CD4^+^ T cells of CD55^-/-^ mice were clearly devoid of CD55 expression at the cell surface (**Figure 4A**) binding of Pra1 was not reduced in the absence of CD55 (**Figure 4B**). Moreover, addition of zinc also increased binding of Pra1 to mouse CD4^+^ T cells of CD55^-/-^ mice (**Figure 4B**). Apart from CD55^-/-^ mice we also studied binding of Pra1 to CD4^+^ T cells of Crry^-/-^ and CD59a^-/-^ mice, which was also not reduced (**Figure 4C**). Therefore, Pra1 does not seem to interact with any of the three complement regulatory proteins expressed on the surface of mouse CD4^+^ T cells.

### Pra1 Binding Co-stimulates Mouse CD4^+^ T Cells

**FIGURE 5:**
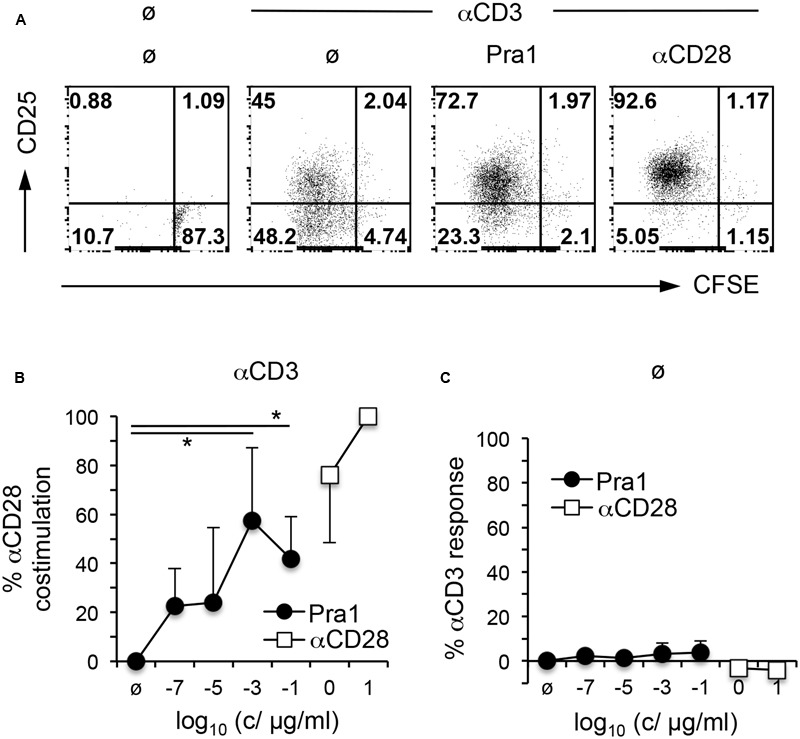
**Pra1 co-stimulates mouse CD4^+^ T cells. (A)** CFSE dilution and CD25 expression by mouse CD4^+^ T cells after a 3 day culture in the presence or absence of plate-bound αCD3 mAb and Pra1 (1 ng/ml) or αCD28 mAb (1 μg/ml) added in solution. **(B)** Summary graph depicting a dose-dependent increase in co-stimulation in the presence of plate-bound αCD3 mAb and Pra1 (0% = frequency CFSE^low^ CD25^+^ with αCD3 mAb only; 100% = frequency CFSE^low^ CD25^+^ with αCD3 + 10 μg/ml αCD28 mAb). **(C)** Direct stimulatory activity of Pra1 in the absence of plate-bound αCD3 (0% = frequency CFSE^low^ CD25^+^ medium only; 100% = frequency CFSE^low^ CD25^+^ with plate-bound αCD3 mAb). **(B,C)**: Means ± SD (*n* = 3 individual experiments). Two-sided *t*-test: ^∗^*p* < 0.05.

To gain further insight into the functional consequences of Pra1 binding to CD4^+^ T cells, we first studied its impact on T cell activation and proliferation *in vitro*. To avoid confounding effects through the interaction of Pra1 with CD11b/CD18 expressed by monocytic cells in our cultures, we FACS-sorted mouse CD4^+^ T cells which lack CD11b/CD18 to more than 99% purity. Stimulation of these highly pure CD4^+^ T cells by plate-bound anti-CD3 mAb and titrated amounts of Pra1 led to a dose-dependent increase in proliferation and CD25 expression similar to what we observed by adding an anti-CD28 mAb (Dennehy et al., 2006) (**Figures 5A,B**). Moreover, Pra1 truly induced a co-stimulatory signal in the T cells as in the absence of CD3 stimulation Pra1 did not activate the cells (**Figure 5C**). The same effect was observed for the anti-CD28 mAb (**Figure 5C**). Binding of Pra1 to mouse CD4^+^ T cells, thus, enhanced T cell activation and proliferation, which comprise the first steps of the adaptive immune response.

### Cytokine Secretion by *In Vivo* Generated Mouse CD4^+^ Memory T Cells is Inhibited in the Presence of Pra1

**FIGURE 6:**
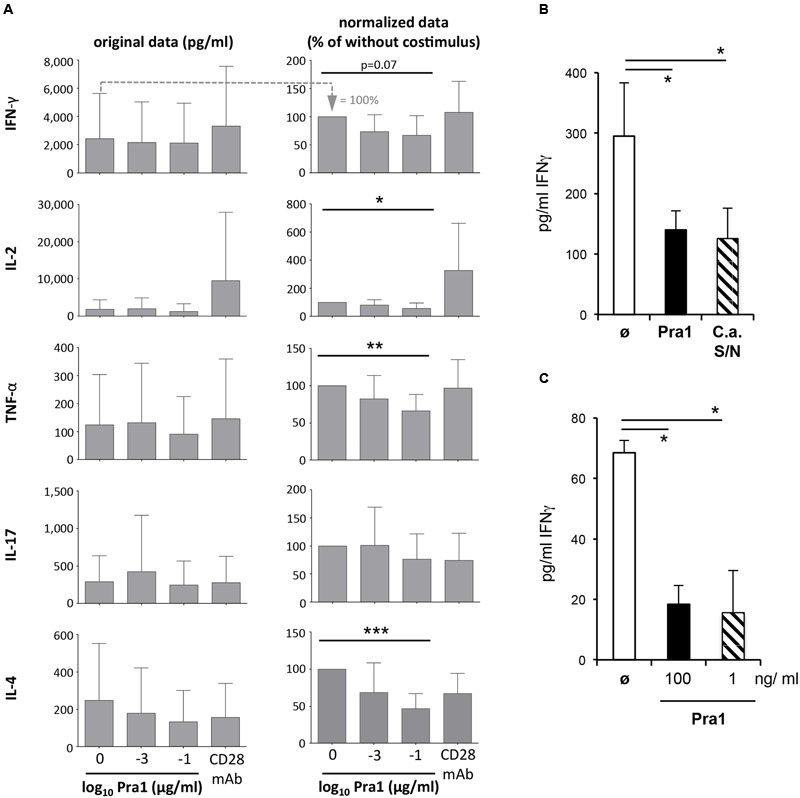
**Modulation of cytokine secretion by Pra1 upon polyclonal and antigen-specific CD4^+^ T cell stimulation. (A)** The left panel shows the absolute amount of cytokines secreted by purified WT CD4^+^ T cells after three days of stimulation with plated-bound anti-CD3-mAb (2.5 μg/ml) either alone or together with Pra1 or anti-CD28 mAb (clone E18, 10 μg/ml) added in solution. The right column shows the amounts of secreted cytokines normalized to the ‘anti-CD3 mAb only’ cultures (=100%; first bar in the left panel). Means + SD of *n* = 5 individual experiments are shown. **(B)** IFNγ secretion upon stimulation of purified CD4^+^ T cells with anti-CD3/anti-CD28 mAb-coated Dynabeads^®^. Pra1 was added at 1 ng/ml and the *C. albicans* supernatant was diluted 1:25. Means + SD of triplicate cultures are shown. The experiment was repeated with similar result. **(C)** Lymph node cells from OT-II mice were stimulated with 1 μM OVAp in the absence or presence of Pra1 as indicated before supernatants were harvested on day three and IFNγ concentrations determined (means ± SD of triplicate cultures; experiment was repeated with similar result). **(A–C)** Two-sided *t*-test. ^∗^*p* < 0.05, ^∗∗^*p* < 0.01, ^∗∗∗^*p* < 0.001.

While the activation of naïve T cells and clonal expansion mark the beginning of the CD4^+^ T cell response, secretion of cytokines such as IFNγ characterize its effector and memory phase. We, therefore, analyzed cytokines in the supernatants of purified CD4^+^ T cells, containing *in vivo* generated memory T cells, stimulated *via* plate-bound anti-CD3 mAb and soluble Pra1 or anti-CD28 mAb (Dennehy et al., 2006) (**Figure 6A**). In contrast to its co-stimulatory effect on T cell activation and proliferation Pra1 suppressed secretion of both Th1 and Th2 cytokines (**Figure 6A**). Only IL-17 secretion appeared not to be affected, while secretion of IL-10 was below the detection limit in these experiments. Seemingly at odds with our observation concerning expression of the IL-2 receptor α-chain, CD25 (**Figure 5**), IL-2 concentrations were also reduced in the presence of Pra1. We assume that this reflects increased IL-2 consumption through increased receptor expression rather than reduced IL-2 production (Malek, 2008) uniting these two findings. To further test the capacity of Pra1 to inhibit cytokine secretion we added Pra1 to purified CD4^+^ T cells which we co-stimulated with anti-CD3/anti-CD28 mAb-coated Dynabeads^®^ (**Figure 6B**). Even under these conditions, which more faithfully mimic T cell-antigen presenting cell interactions than stimulation *via* plate-bound antibodies, Pra1 reduced cytokine, i.e., IFNγ, secretion by the CD4^+^ T cells (**Figure 6B**). The same was true for the supernatant of cultured *C. albicans* containing the whole array of secreted fungal proteins (**Figure 6B**).

Apart from binding to CD4^+^ T cells, Pra1 interacts with CD11b/CD18 integrin (Mac1) expressed by monocytic and granulocytic cells (Soloviev et al., 2011). To test whether Pra1 also suppresses IFNγ secretion in the presence of Mac1-expressing antigen-presenting cells (APCs) we stimulated total splenocytes from T cell receptor-transgenic OT-II mice with 1 μM OVA-peptide 323–339 in the presence of 100 or 1 ng/ml Pra1 (**Figure 6C**). Also under these conditions Pra1 inhibited IFNγ secretion by the OT-II CD4^+^ T cells.

Both in the presence of recombinant Pra1 as well as *C. albicans* supernatant, secretion of cytokines by mouse CD4^+^ T cells was, thus, reduced.

### Depending on the Strength of the TCR Signal Pra1 Also Reduces Secretion of IFNγ by *In Vitro* Generated Th1 Cells

**FIGURE 7:**
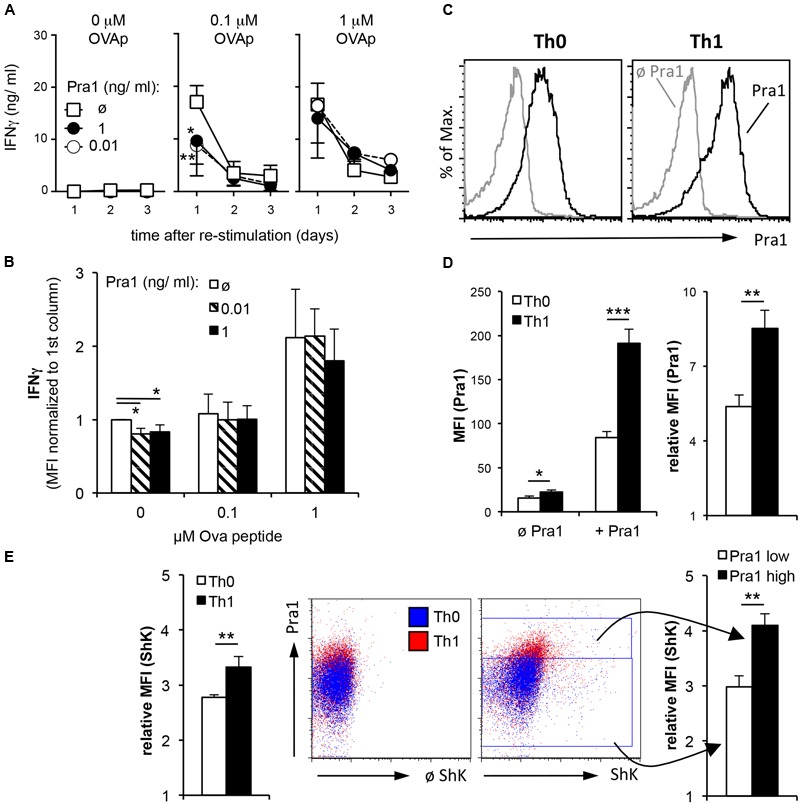
**IFNγ secretion by OT-II Th1 cells in the presence of Pra1. (A)** *In vitro* generated OT-II Th1 cells were re-stimulated in the presence of the indicated amounts of OVAp, Pra1 and APCs. Culture supernatants were harvested on days one to three and IFNγ content determined. Means ± SD of triplicate cultures are shown. The experiment was repeated with similar result. An ANOVA was used to compare cultures with to those without Pra1. **(B)** At the end of the re-stimulation cultures, we determined IFNγ expression by OT-II Th1 cells intracellularly. Means ± SD of three independent experiments are shown. A two-sided *t*-test was used to compare groups. **(C)** Representative Pra1 staining (black; gray: control without Pra1) of OT-II CD4^+^ T cells stimulated with OVAp under Th0 (left; 1% IFNγ^+^) or Th1 conditions for 5 days (right, 53% IFNγ^+^). **(E)** Detection of Kv1.3 expression via staining in parallel to Pra1 binding in the samples shown in **(C,D)**. **(D,E)**: Means ± SD of triplicate analyses. The experiment was repeated twice with similar result. A two-sided *t*-test was used to compare groups. ^∗^*p* < 0.05, ^∗∗^*p* < 0.01, ^∗∗∗^*p* < 0.001.

T cells isolated from healthy mice producing effector cytokines are by definition mostly resting memory T cells. During acute invasive *C. albicans* infection or *C. albicans*-induced inflammation the fungus, however, mainly encounters effector T cells. Therefore, we first deliberately generated OT-II Th1 effector cells during a five-day culture *in vitro* followed by a two-day resting phase and subsequent re-stimulation of the Th1 cells in the presence of APCs and different concentrations of peptide antigen and Pra1 (**Figure 7A**). Addition of Pra1 to Th1 cells stimulated with 0.1 μM OVA peptide reduced IFNγ secretion into the supernatant (**Figure 7A**, middle), while this was not the case at 1 μM OVA peptide (**Figure 7A**, right). Analyzing intracellular IFNγ expression by the Th1 cells after PMA/ionomycin re-stimulation, further, showed that the reduced secretion of IFNγ into the culture supernatant in the presence of Pra1 was not due to a per se lower capacity of the Th1 cells to produce IFNγ. Without OVAp re-stimulation the expression of IFNγ by the Th1 cells was, however, reduced suggesting that Pra1 increases the threshold for stimulation-induced cytokine secretion by CD4^+^ T cells. Incubation of Th1 cells with Pra1 showed, in comparison to OT-II CD4^+^ T cells cultured under Th0 conditions in parallel, that Th1 cells bind Pra1 better than Th0 cells (**Figures 7C,D**) suggesting that differentiated effector memory Th1 cells are a primary target of Pra1. In autoreactive pathogenic T cells Kv1.3 has been shown to be the main voltage-gated potassium channel and blocking the channel with the ShK peptide inhibits the autoreactive pathogenic T cells in animal models of autoimmunity *in vivo* (Beeton et al., 2001) and in cell cultures of human T cells *in vitro* (Wulff et al., 2003). Using a fluorescently labeled ShK peptide we observed that the Th1 cells expressed more Kv1.3 channels than Th0 cells and that Pra1 binding and Kv1.3 expression were positively correlated in Th1 cells (**Figure 7E**). Pra1, thus, preferentially bound to effector/memory Th1 cells inhibiting IFNγ secretion provided TCR stimulation did not surpass a certain threshold.

## Discussion

In this study, we describe the direct interaction of the secreted *C. albicans* protein Pra1 with mouse CD4^+^ T cells. Binding of Pra1 to the CD4^+^ T cells was enhanced by extracellular Zn^2+^. Moreover, Pra1 binding inhibited cytokine secretion from CD4^+^ T cells *in vitro* thus constituting a novel immune evasion mechanism for *C. albicans*.

In line with its known capacity to scavenge Zn^2+^ ions (Citiulo et al., 2012) Pra1 bound more efficiently to mouse CD4^+^ T cells in the presence of extracellular zinc than in its absence (**Figure 1**). This activity was in contrast to what we observed for Aspf2, the zinc-binding Pra1-homolog of *A. fumigatus* (Citiulo et al., 2012). Aspf2 did not bind to mouse CD4^+^ T cells – either in the presence or absence of Zn^2+^ (**Figure 3**). As both proteins carry a HIS-tag, which, of course, by itself is capable of binding Zn^2+^ (Evers et al., 2008), the enhanced binding of Pra1 to mouse CD4^+^ T cells after addition of ZnCl_2_ was not merely mediated by the HIS-tag. Moreover, even under conditions where we did not add ZnCl_2_ during the staining procedure we detected a positive signal for Pra1 binding (**Figures 1, 2**). This data implies that the Pra1 binding to the surface of the CD4^+^ T cells is not strictly zinc-dependent and/or that free zinc present in preparations of lymph node cells and splenocytes might be sufficient to allow for Pra1 binding.

The molecular basis for the enhanced Pra1 binding mediated by ZnCl_2_ is so far not clear. We envisage that Zn^2+^ binding might induce a conformational change in Pra1 as has been described for many other Zn^2+^-binding proteins (Ebert and Altman, 2008). Such a structural change has, however, not yet been described for Pra1.

While the receptor for Pra1 on the surface of mouse CD4^+^ T cells is still elusive, CR3 (CD11b/CD18, Mac-1) expressed by neutrophils and monocytic cells has been shown to bind Pra1 and that this binding is important to protect mice after systemic *C. albicans* infection (Soloviev et al., 2011). On mouse CD4^+^ T cells it is, however, not a complement regulatory protein that interacts with Pra1 (**Figure 4**). Therefore, it is unlikely that modulation of complement activation, which has been shown to crucially contribute to T cell stimulation and differentiation (Arbore and Kemper, 2016), accounts for the effects of Pra1 on mouse CD4^+^ T cells. Analysis of Kv1.3 expression in parallel to Pra1 binding to *in vitro* polarized CD4^+^ Th1 cells, however, showed that cells with the highest capacity to bind Pra1 also expressed high levels of Kv1.3 (**Figure 7**). While we do not, yet, know whether Kv1.3 is a receptor for Pra1 it may not be the only molecule Pra1 interacts with on the T cell surface. Kv1.3 expression cannot be detected on resting T cells by FACS using the ShK-F6CA peptide (Beeton et al., 2003), while Pra1 binding to resting T cells is detectable by flow cytometry as detailed in this study. Functionally, Pra1 might interfere with K^+^ currents through Kv1.3 by direct binding to the channel or by binding in the vicinity of Kv1.3 and ‘delivering’ Zn^2+^ ions. Extracellular Zn^2+^ binds to Kv1.3 inhibiting the transport of K^+^ ions through the channel (Teisseyre and Mozrzymas, 2002, 2006).

Apart from directly interacting with CD4^+^ T cells, Pra1 could also modulate T cell responses by binding to APCs *via* interaction with CD11b/CD18 (Soloviev et al., 2011) or *via* the still unknown Pra1 receptor also expressed on T cells. Therefore, it was important to study the effects on cytokine secretion by CD4^+^ T cells in the presence of APCs. Irrespective of whether APCs were present in our assays Pra1 inhibited cytokine secretion by the CD4^+^ T cells (**Figures 6, 7**) suggesting that the direct interaction of Pra1 with the CD4^+^ T cells was also the crucial event in the cultures containing APCs.

Recombinant Pra1 and supernatant of *C. albicans* cultures inhibited IFNγ release from CD4^+^ T cells (**Figure 6**). While we do not know to what extent Pra1 contributes to the overall inhibitory effect of the *C. albicans* supernatant this observation highlights that *C. albicans*, through its secretome, modulates CD4^+^ T cell responses. Further experimentation is required to delineate whether Pra1 is the only *C. albicans* protein mediating these effects or, more likely, whether other secreted fungal proteins also contribute to effector T cell inhibition.

Apart from Th1 cells, Th17 cells also crucially contribute to anti-fungal immunity either through direct effects or by supporting Th1 versus Th2 cell differentiation (Romani, 2011; Zelante et al., 2016). In contrast to other cytokines, IL-17 release from CD4^+^ T cells was not reduced in the presence of Pra1 (**Figure 6**). This might have to do with the degree of TCR signal strength required to induce optimal cytokine release from different CD4^+^ T helper cell subpopulations. For Th1 cells we observed that strong TCR stimulation overcame Pra1-induced suppression of cytokine release (**Figure 7**). As maximal IL-17 release, in contrast to IFNγ release, has been reported to require low TCR stimulation (Purvis et al., 2010) further experimentation is required to determine whether, indeed, Pra1 differentially regulates cytokine release from Th1 and Th17 cells.

In summary, our data identify Pra1 as an inhibitor of mouse CD4^+^ effector T cell function *in vitro*, thus, mediating evasion of *C. albicans* from potentially harmful CD4^+^ T cell responses. While subversion of the CD4^+^ T cell response during commensalism might be of mutual benefit for *C. albicans* and the host, during invasive infection/sepsis blocking protective CD4^+^ T cell immunity might worsen clinical outcome. Therefore, the findings of our study suggest that therapeutic targeting of soluble Pra1 might enhance CD4^+^ T cell responses protecting the host from invasive *C. albicans* infections.

## Author Contributions

AB designed research studies, conducted experiments, acquired and analyzed data, and wrote the paper. PD provided reagents, designed research studies, and interpreted data. SW conducted experiments, acquired and analyzed data. TRH provided reagents, designed research studies, and interpreted data. WS provided reagents and interpreted data. PH provided reagents and designed research studies. AB provided reagents, designed research studies, and interpreted data. TH designed research studies and analyzed and interpreted data. PZ provided reagents, designed research studies, interpreted data, and wrote the manuscript. NB designed research studies, analyzed, and interpreted data and wrote the paper.

## Conflict of Interest Statement

The authors declare that the research was conducted in the absence of any commercial or financial relationships that could be construed as a potential conflict of interest.
